# H2A.Z landscapes and dual modifications in pluripotent and multipotent stem cells underlie complex genome regulatory functions

**DOI:** 10.1186/gb-2012-13-10-r85

**Published:** 2012-10-03

**Authors:** Manching Ku, Jacob D Jaffe, Richard P Koche, Esther Rheinbay, Mitsuhiro Endoh, Haruhiko Koseki, Steven A Carr, Bradley E Bernstein

**Affiliations:** 1 Department of Pathology, Center for Systems Biology and Center for Cancer Research, Massachusetts General Hospital, Simches Research Building, CPZN 8234, Boston, MA 02114, USA; 2 Howard Hughes Medical Institute, 4000 Jones Bridge Road, Chevy Chase, MD 20815-6789, USA; 3 Broad Institute of Harvard and Massachusetts Institute of Technology, 7 Cambridge Center, Cambridge, MA 02142, USA; 4 Department of Pathology, Harvard Medical School, 77 Avenue Louis Pasteur, Boston, MA 02115, USA; 5 Division of Health Sciences and Technology, Massachusetts Institute of Technology, 77 Massachusetts Avenue, E25-519, Cambridge, MA 02139, USA; 6 Bioinformatics Program and Department of Biomedical Engineering, Boston University, 24 Cummington Street, Boston, MA 02215, USA; 7 RIKEN Research Center for Allergy and Immunology, 1-7-22 Suehiro-cho, Tsurumi-ku, Yokohama, Kanagawa 230-0045, Japan

**Keywords:** Acetylation, bivalent, ChIP-Seq, H2A.Z, mass spectrometry; ubiquitination

## Abstract

**Background:**

The histone variant H2A.Z has been implicated in nucleosome exchange, transcriptional activation and Polycomb repression. However, the relationships among these seemingly disparate functions remain obscure.

**Results:**

We mapped H2A.Z genome-wide in mammalian ES cells and neural progenitors. H2A.Z is deposited promiscuously at promoters and enhancers, and correlates strongly with H3K4 methylation. Accordingly, H2A.Z is present at poised promoters with bivalent chromatin and at active promoters with H3K4 methylation, but is absent from stably repressed promoters that are specifically enriched for H3K27 trimethylation. We also characterized post-translational modification states of H2A.Z, including a novel species dually-modified by ubiquitination and acetylation that is enriched at bivalent chromatin.

**Conclusions:**

Our findings associate H2A.Z with functionally distinct genomic elements, and suggest that post-translational modifications may reconcile its contrasting locations and roles.

## Background

Pluripotent embryonic stem (ES) cells are characterized by a plastic epigenome conducive to self-renewal and broad differentiation potential. Histones and chromatin proteins in ES cells are subject to relatively rapid turnover [1-3]. This dynamic exchange is thought to maintain an accessible and transcriptionally competent state [4,5]. During development, this initially permissive chromatin configuration becomes restricted as cells progressively commit to specific lineages.

Pluripotent chromatin is distinguished by characteristic post-translational histone modifications. Bivalent domains that contain 'active' H3 lysine 4 trimethylation (H3K4me3) and 'repressive' H3 lysine 27 trimethylation (H3K27me3) are prevalent in ES cells. Bivalent domains and associated Polycomb repressive complexes 1 and 2 (PRC1 and PRC2) silence developmental loci while maintaining their potential for future activation [2]. In fact, some of these loci may already be engaged by initiating RNA polymerase II (RNAPII) [6]. During lineage specification, bivalent domains often resolve into monovalent domains enriched for either modification in accordance with gene expression. Developmental genes that are not expressed within the relevant lineage often retain H3K27me3 domains [7].

Replication-independent histone deposition is of particular interest as it is targeted to DNA sequences under active regulation [8,9]. Rapid nucleosome turnover is a general feature of promoters and epigenetic regulatory elements in yeast [10] and in fly [11]. In flies and mammals, nucleosome-exchange hotspots, including promoters, sites of transcriptional initiation and transcription factor (TF) binding sites, are also enriched for the histone variant H3.3 [12]. In mammals, H3.3 can coexist with H2A.Z in the same nucleosome, and these double-variant-containing nucleosomes represent the most labile fraction of the accessible active promoters, enhancers and putative insulators [13]. H2A.Z, an evolutionarily conserved H2A variant, has been implicated in multiple functions. H2A.Z localizes to transcription start sites (TSSs) where it frequently flanks nucleosome-deficient regions [14,15]. This variant is also associated with other genomic sites undergoing histone exchange, including intergenic CCCTC-binding factor (CTCF) binding sites in mammals and boundary elements in yeast [8,13,15]. H2A.Z-containing nucleosomes are unusually susceptible to nuclease digestion and stringent ionic conditions [16,17], and it has been speculated that this structural instability is because of amino acid substitutions at the interface between H2A.Z and H3/H4 [18]. Overall, these findings suggest that H2A.Z indexes genomic regions of specific regulatory functions for rigorous nucleosome disassembly and reassembly. That this variant is also essential for mammalian development reinforces the significance of chromatin dynamics to genome regulation [19,20].

In addition to its pervasive roles at TSSs and active regulatory elements, H2A.Z has also been linked to Polycomb regulation. A microarray-based chromatin immunoprecipitation (ChIP-chip) analysis in ES cells found that H2A.Z associates exclusively with silent promoters bound by PRC2 [21]. Upon differentiation, H2A.Z was found to relocate to active TSSs. These findings suggested that H2A.Z plays a distinct role in ES cells that is tightly linked to Polycomb repression. However, this study relied primarily on promoter microarrays that are not comprehensive [15], and antibody reagents that may not account for potential modifications [22]. Moreover, the findings are not entirely consistent with those of H2A.Z studies carried out in other cell models and in other organisms.

To clarify the distribution and potential functions of H2A.Z in ES cells, we used ChIP coupled with high-throughput sequencing (ChIP-Seq) to query the localization of this variant in mouse and human ES cells, and in lineage-restricted progenitors. We found that H2A.Z is ubiquitously deposited at promoters, putative enhancers and other intergenic regulatory elements marked by H3K4 methylation. H2A.Z is also deposited at K27me3 regions/PRC2 binding sites, but it is restricted to those sites that have coexisting H3K4 methylation, and thus constitute bivalent domains. Notably, we found that bivalent chromatin is enriched for a novel population of H2A.Z simultaneously modified by N-terminal acetylation and C-terminal ubiquitination. We propose that distinct modification states enable H2A.Z to facilitate regulation of bivalent PRC2 targets as well as to act at a diversity of other histone exchanging elements in mammalian genomes.

## Results and discussion

### Genomewide H2A.Z enrichment at active and bivalent promoters

**Figure 1:**
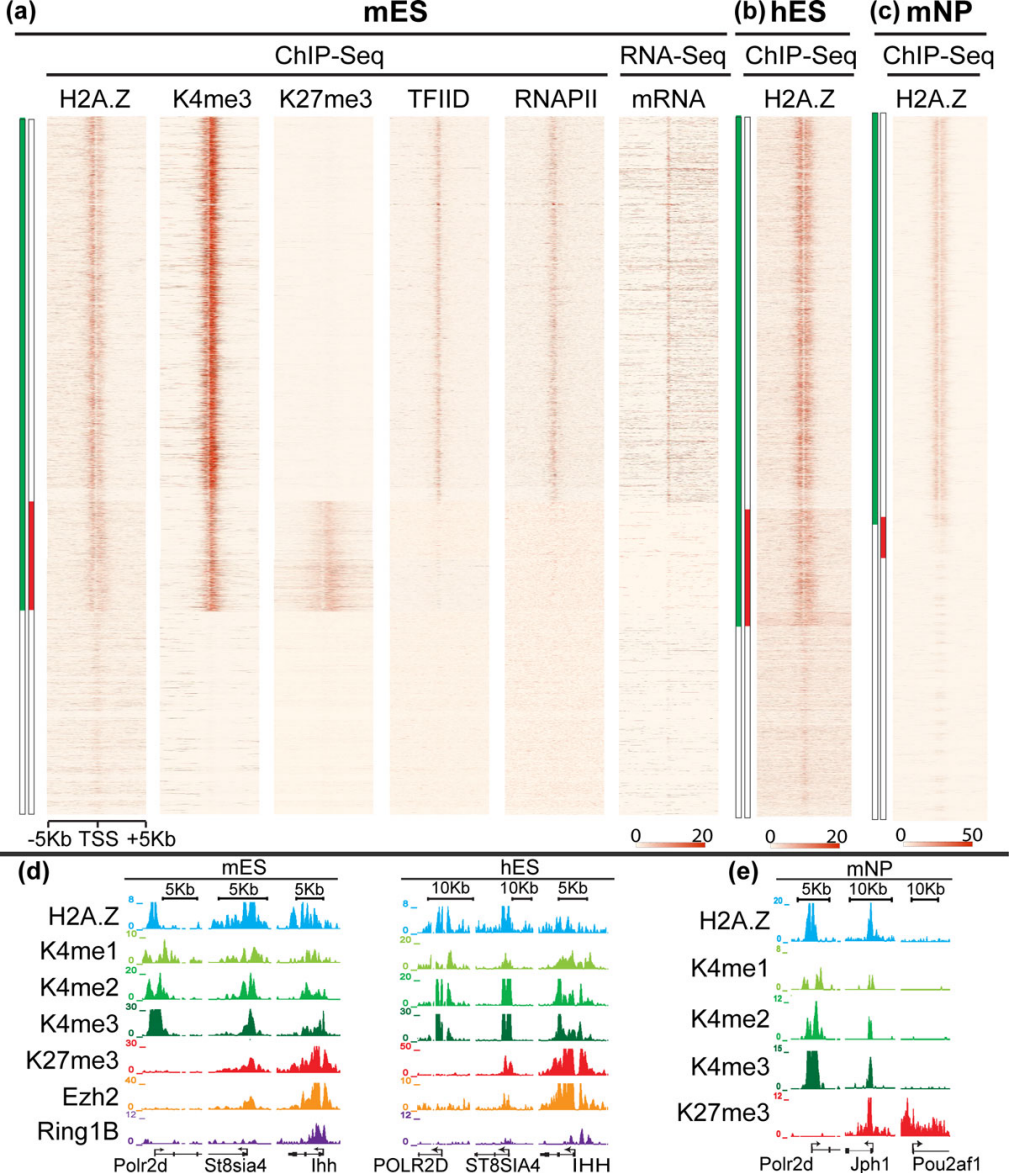
**H2A.Z localizes to promoters in embryonic stem cells and neural progenitor cells**. **(a) **Heatmaps depict H2A.Z, H3K4me3, H3K27me3, TFIID and RNAPII ChIP-Seq and RNA-Seq signals at regions spanning all transcription start sites (±5 kb) in mES cells organized according to their chromatin status: H3K4me3 (green), H3K27me3 (red), bivalent (green and red), and no-mark (white). Active K4me3-only (green only) promoters show strong signals for H2A.Z, transcriptional machinery (TFIID, RNAP II) and mRNA, while bivalent promoters (green and red) are primarily enriched for H2A.Z. **(b) **Analogous heatmap for H2A.Z in human embryonic stem (hES) cells. H2A.Z occupancy at active and bivalent promoters is conserved between mES and hES cells. **(c) **Analogous heatmap for H2A.Z in mouse neural progenitor (mNP) cells. H2A.Z signal is depleted from monovalent K27me3-only promoters (red, in the absence of green). **(d) **ChIP-Seq tracks show H2A.Z localization to K4me3-only (Pol2rd) and bivalent promoters (St8sia4 and Ihh) in mES and hES cells. **(e) **ChIP-Seq tracks show H2A.Z localizes to K4me3-only (Pol2rd) and bivalent promoters (Jph1) in mNP cells, but not monovalent H3K27me3-only (Pou2af1) promoters. ChIP-Seq: chromatin immunoprecipitation coupled with high-throughput sequencing; hES: human embryonic stem; H3: histone H3 K: lysine; kb: kilobase; me1: monomethylation; me2: dimethylation; me3: trimethylation; mES: mouse embryonic stem; mNP: mouse neural progenitor; RNAPII: RNA polymerase II; TFIID: transcription factor IID; mRNA: messenger RNA.

**Figure 2:**
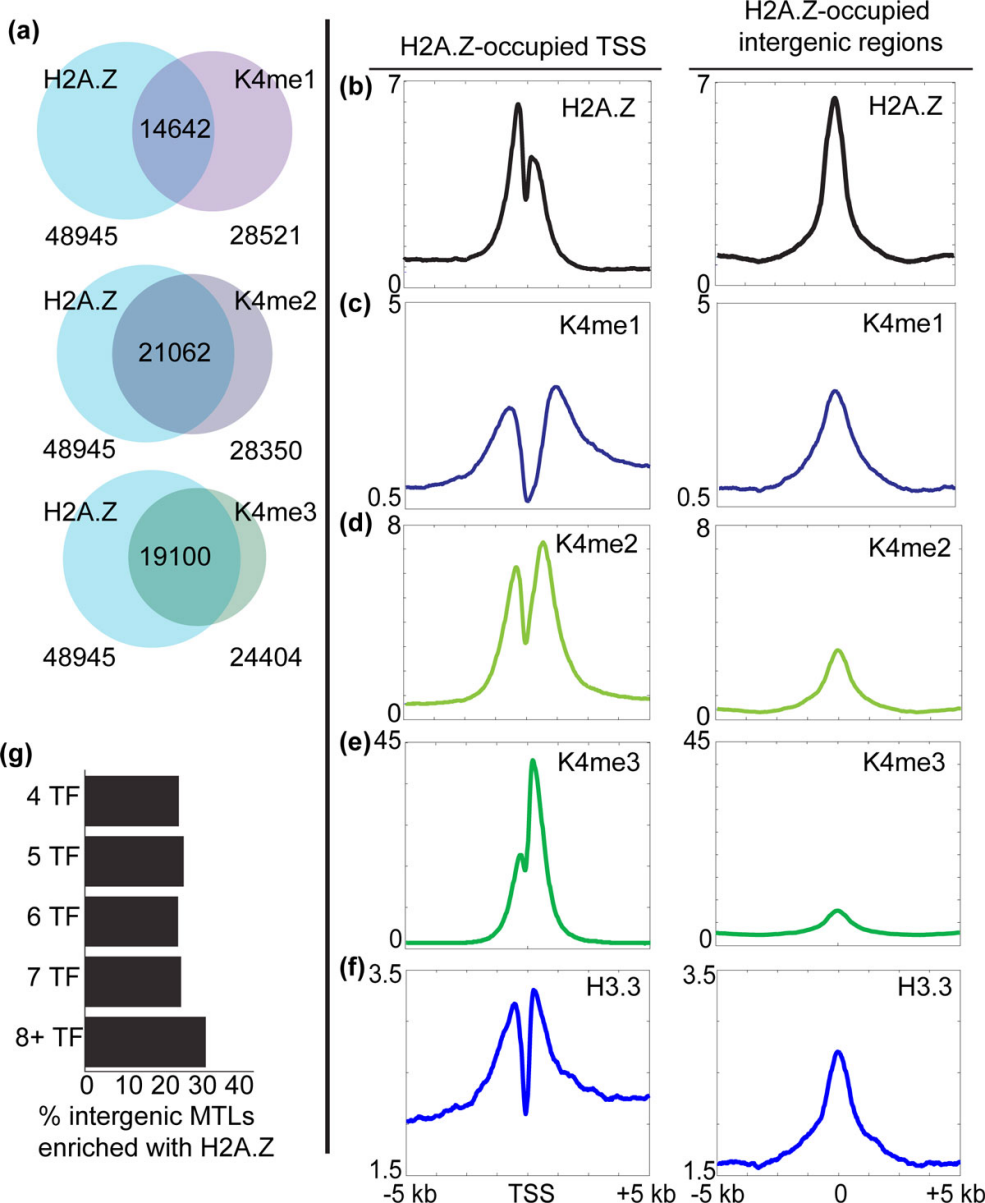
**H2A.Z highly correlates with H3K4 methylation and localizes to distal elements**. **(a) **Venn diagrams show overlaps of H2A.Z-enriched genomewide intervals with H3K4me1, H3K4me2 and H3K4me3. **(b) **Composite plots of ChIP-Seq signals for H2A.Z across TSSs (±5 kb, left panel) and intergenic sites (±5 kb, right panel) enriched for H2A.Z. **(c) **Analogous composite plots showing enrichment of H3K4me1, a putative enhancer mark, at intergenic H2A.Z sites. **(d) **Analogous composite plots for H3K4me2, which shows enrichment in both TSSs and enhancers. **(e) **Analogous composite plots for H3K4me3, which is enriched at H2A.Z-occupied TSSs. **(f) **Analogous composite plots for histone variant H3.3, which is enriched at H2A.Z-occupied TSSs and enhancers. **(g) **Bar graph shows fractions of intergenic MTLs [28] occupied by H2A.Z in mES cells, suggesting the correlation between H2A.Z and regulatory genomic regions under nucleosome regulation. ChIP-Seq: chromatin immunoprecipitation coupled with high-throughput sequencing; H3: histone H3, K: lysine; kb: kilobases; me1: monomethylation; me2: dimethylation; me3: trimethylation; MTLs: multiple transcription factor binding loci; TF: transcription factor; TSSs: transcription start sites; mES: mouse embryonic stem.

We acquired genome-wide maps of H2A.Z in mouse ES (mES) cells using ChIP-Seq. We observed H2A.Z enrichment at a substantial majority of TSSs, with a bimodal distribution spanning the promoters and the 5' end of TSSs specifically (Figure 1a, H2A.Z; Figure 1d, mES cells; Figure 2b). This is concordant with previous observations that H2A.Z flanks and is enriched around the nucleosome-deficient regions of the TSS itself. We compared three major classes of TSSs in ES cells, 'H3K4me3-only', 'bivalent' (H3K4me3 and H3K27me3), and 'no-mark' (both marks are absent) [7,23]. Bivalent, PRC2-target promoters are strongly enriched for H2A.Z, as reported (Figure 1a,d, mES cells) [21]. However, H2A.Z also showed comparable enrichment levels at essentially all H3K4me3-only promoters [24]. By contrast, no-mark TSSs have very low or no H2A.Z enrichment.

### Principles of H2A.Z occupancy conserved between species and cell states

We used the same ChIP-Seq procedures and antibody to profile H2A.Z in human ES (hES) cells (Figure 1b,d, hES cells). We again compared bivalent, H3K4me3-only and no-mark promoters, from previously described hES cell ChIP-Seq data [25]. The H2A.Z signal in hES cells shows a strong bimodal pattern of intensity at H3K4me3-only and bivalent TSSs (Figure 1b,d, hES cells; Figure 2b); an identical promoter architecture to that observed in mES cells (Figure 1a). Next, we examined mouse neural progenitors (mNPs), where H2A.Z again localizes to active promoters as previously reported for differentiated cell types (Figure 1c,e). H2A.Z is also enriched at some PRC2 target promoters in mNPs, but is limited to the subset of Polycomb targets that also carries H3K4me3. Together, these findings suggest that H2A.Z patterns are conserved among species and between cell types, and that co-localization of H2A.Z with PRC2 binding sites reflects coexisting active chromatin modifications (Figure 1c). Furthermore, the data suggest that progression of certain PRC2 target loci from bivalent to H3K27me3-only during differentiation is accompanied by marked reductions in the accessibility of chromatin, which might underlie a more stably repressed chromatin state.

### H2A.Z correlates with alternate H3K4 methylation states at promoters and enhancers

Further evidence for correlation between H2A.Z and H3K4 methylation emerges from genome-wide analyses of H3K4 monomethylation (me1), dimethylation (me2) and trimethylation (me3). Respectively, 51% of H3K4me1-, 74% of H3K4me2- and 78% of H3K4me3-enriched regions overlap with H2A.Z sites (Figure 2a). H2A.Z enriches at similar level in promoters, as it does in identified intergenic regions (Figure 2b). H3K4me1, which is enriched at enhancers, strongly associates with intergenic H2A.Z (Figure 2c) [26]. As previously described, H3K4me2 is enriched at both promoters and enhancers (Figure 2d), while H3K4me3 predominantly marks promoters (Figure 2e) [26]. In all H3K4 methylation contexts, H2A.Z was suitably enriched. The localization of H2A.Z in ES cells is also associated with the presence of histone H3 variant, H3.3, which has been linked to replication-independent deposition [12]. Indeed, we observed relative enrichment of H3.3 at H2A.Z-positive enhancers and promoters (Figure 2f, Additional file 1). H2A.Z also occupies between 20% and 30% of multiple TF binding loci (MTLs) (Figure 2g), supporting its localization at accessible chromatin [26-29]. Together, the data suggest a high degree of correspondence between H2A.Z and sites of euchromatin in ES cells.

### Promoter H2A.Z sites flank nucleosome-deficient regions enriched for transcriptional machinery

These observations led us to consider the basis for the presence of H2A.Z and associated nucleosome replacement with histone variants at bivalent PRC2 target sites in ES cells. We therefore acquired and analyzed ChIP-Seq maps for the pre-initiation complex component TFIID (transcription Factor II D), hypo-phosphorylated RNAPII and pan-histone H3. We also incorporated published RNA-Seq data for mES cells [7,30]. Integrative analyses across different classes of TSSs reveal strong enrichment for RNAPII and TFIID over the nucleosome-deficient regions of H3K4me3-only TSSs (Figure 1a, TFIID, RNAPII). As expected, RNA transcripts are also strongly represented across this class of TSSs (Figure 1a, mRNA). By contrast, promoters lacking H3K4me3 (no-mark) show essentially no signal for RNAPII, TFIID or RNA transcripts (Figure 1a, mRNA). PRC2-bound bivalent genes exhibit subtle enrichments for TFIID and, to a lesser extent, for RNAPII, but no productive mRNA is observed. These data suggest that bivalent TSSs are accessible to the transcriptional apparatus, although the associations are likely transient. ChIP-Seq analysis of pan-histone H3 shows that clearly defined nucleosome-deficient regions are present at K4-only and bivalent promoters, but not at no-mark promoters (Additional file 2). These activities might be sufficient to promote and maintain nucleosome-deficient regions and facilitate H2A.Z turnover at bivalent TSSs to retain their transcriptional competence. Alternatively, the chromatin patterns could reflect yet undefined sequence determinants that directly recruit chromatin regulators and RNAs, or innately destabilize nucleosomes [31,32].

**Figure 3:**
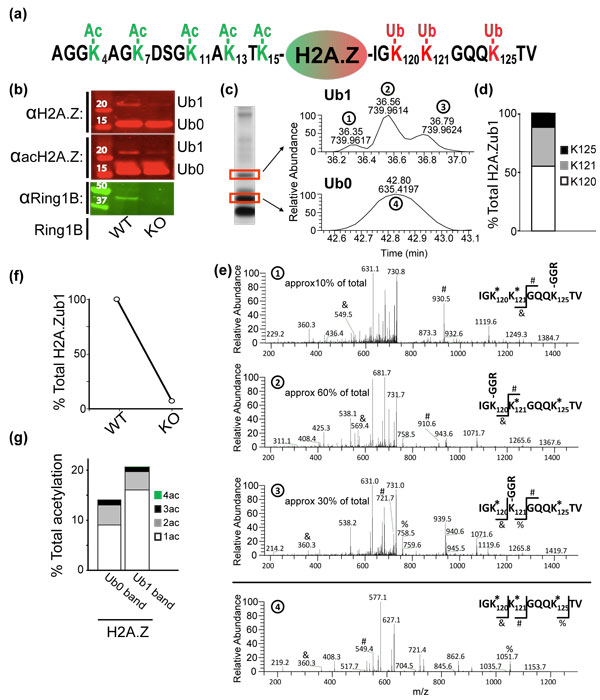
**A dually modified H2A.Z species bearing ubiquitination and acetylation**. **(a) **Schematics of H2A.Z N-terminal and C-terminal amino acid sequences showing lysine residues that are can be acetylated (Ac) or monoubiquitinated (Ub1) according to MS analysis. **(b) **Western blots for H2A.Z (top panel), acH2A.Z (middle panel) and Ring1B (bottom panel) for control (left lane) and tamoxifen-induced Ring1B KO (right lane) mES cells. The data suggest that the PRC1 component Ring1B is upstream of H2A.Zub1 and acH2A.Zub1 in mES cells. **(c) **Left: SDS-PAGE shows separation of HPLC-purified H2A.Zub1 (top band) from other H2A.Z species (bottom band). Red boxes indicate bands excised for MS analysis. Right: Extracted ion chromatograms of the excised bands show H2A.Zub1 is present in the upper band and is absent in the lower band. *m/z *values indicate the residual of H2A.Zub1 after *d_5_*-propionylation and chymotryptic digestion (upper trace). **(d) **Quantification of the C-terminal H2A.Z monoubiquitination at K120, K121 and K125 residues in mES cells. **(e) **MS/MS spectra assign the positional isomers of ubiquitination (Ub1) in each peak from Figure 3c, right panel ((1), (2), (3) and (4) respectively). The three peaks in the upper trace in Figure 3c correspond to differential sites of H2A.Zub1. (-GGR) sites indicate the branched peptide that results from monoubiquitin ligation at a given residue followed by chymotryptic digestion. * indicates d5-propionylation and therefore an absence of ubiquitin at a given lysine. (#,&,%) marks indicate key ions that localize the site of ubiquitination on the peptide. **(f) **Abundance of C-terminal ubiquitination of H2A.Z in wild type and Ring1B KO mES cells. Signal corresponds to area under the peak(s) observed by MS corresponding to the H2A.Z 118-127 peptide with one ubiquityl adduct, as in Figure 3c. **(g) **Quantitative MS analysis shows the prevalence of one acetylation (1ac), two acetylation (2ac), three acetylation (3ac) and four acetylation (4ac) species on any N-terminal lysines within non-ubiquitinated and ubiquitinated H2A.Z populations separated by SDS-PAGE. Despite its apparently repressive function, H2A.Zub1 is more frequently acetylated. Ac: acetylated; HPLC: high performance liquid chromatography; KO: knockout; mES: mouse embryonic stem cells; MS: mass spectrometry; PRC1: Polycomb repressive complex 1; Ub0: non-ubiquitinated; Ub1: ubiquitinated; WT: wild type.

The presence of H2A.Z at varied classes of genomic elements is consistent with its diverse functions in transcriptional initiation and induction; regulation of DNA methylation; and Polycomb repression [21,29,33]. However, since the different genomic elements are packaged in markedly different chromatin contexts, we considered whether H2A.Z might exhibit differential post-translational modifications in these different contexts. We therefore examined two specific modifications of H2A.Z: acetylation and ubiquitination (Figure 3a) [22,34].

### Monoubiquitination of H2A.Z is downstream of Ring1B

H2A.Z has been shown to be subject to C-terminal ubiquitination by the PRC1 component Ring1B [22]. Western blots performed on acid-extracted histones from mES cells with antibody against H2A.Z revealed the presence of the native variant as well as an additional species (approximately 22 kDa) whose molecular weight is consistent with the addition of one ubiquitin moiety (Figure 3b, anti-H2A.Z: Ub0 and Ub1 band). Mass spectrometry (MS) analysis confirmed that the majority of H2A.Z in the higher molecular weight species contains the residual adduct of a ubiquitin distributed amongst K120 (>60%), K121 (approximately 30%) and K125 (approximately 10%) (Figure 3c,d,e). The observation of multiple ubiquitin acceptor sites is consistent with prior reports, and may reflect promiscuity of the E3 ligase [22]. We then tested whether all sites depend on the PRC1 component Ring1B. The monoubiquitination levels of all three lysines are dramatically reduced in Ring1B knockout mES cells according to western blot and MS analyses (Figure 3b, right panel; 3f). While some redundancy or slow turnover of non-ubiquitinated species may account for residual ubiquitinylation in our system, our MS data clearly confirm Ring1B as the principal ubiquitin E3 ligase for all sites on the H2A.Z C-terminus.

### A dually modified H2A.Z species with ubiquitination and acetylation

We next explored the relationship between C-terminal ubiquitination and N-terminal acetylation on H2A.Z. Although prior studies have linked H2A.Z acetylation to transcriptional activity [34-37], western blot analysis showed that the anti-acetylated-H2A.Z antibody also recognizes the '+1 ubiquitin' species (Figure 3b, anti-acH2A.Z, Ub1 band). MS analysis further supports the co-occurrence of the two types of H2A.Z modifications on the same molecule. Because the +Ub1 shifts H2A.Z significantly on SDS-PAGE analysis, we were able to isolate individual H2A.Zub0 and H2A.Zub1 bands respectively. We performed MS analyses on these isolated bands and confirmed that the H2A.Zub1 band is essentially all ubiquitinated (Figure 3d). Furthermore, within the H2A.Zub1 fraction, MS analysis specifically shows that approximately 21% of H2A.Zub1 is acetylated, indicating that about one fifth of the H2A.Zub1 population carries ubiquitination and acetylation concurrently (Figure 3g). Remarkably, quantitative MS analysis also indicates that H2A.Zub1 species have higher levels of N-terminal acetylation relative to their non-ubiquitinated counterpart, but possess differential acetylation profiles (Figure 3g).

**Figure 4:**
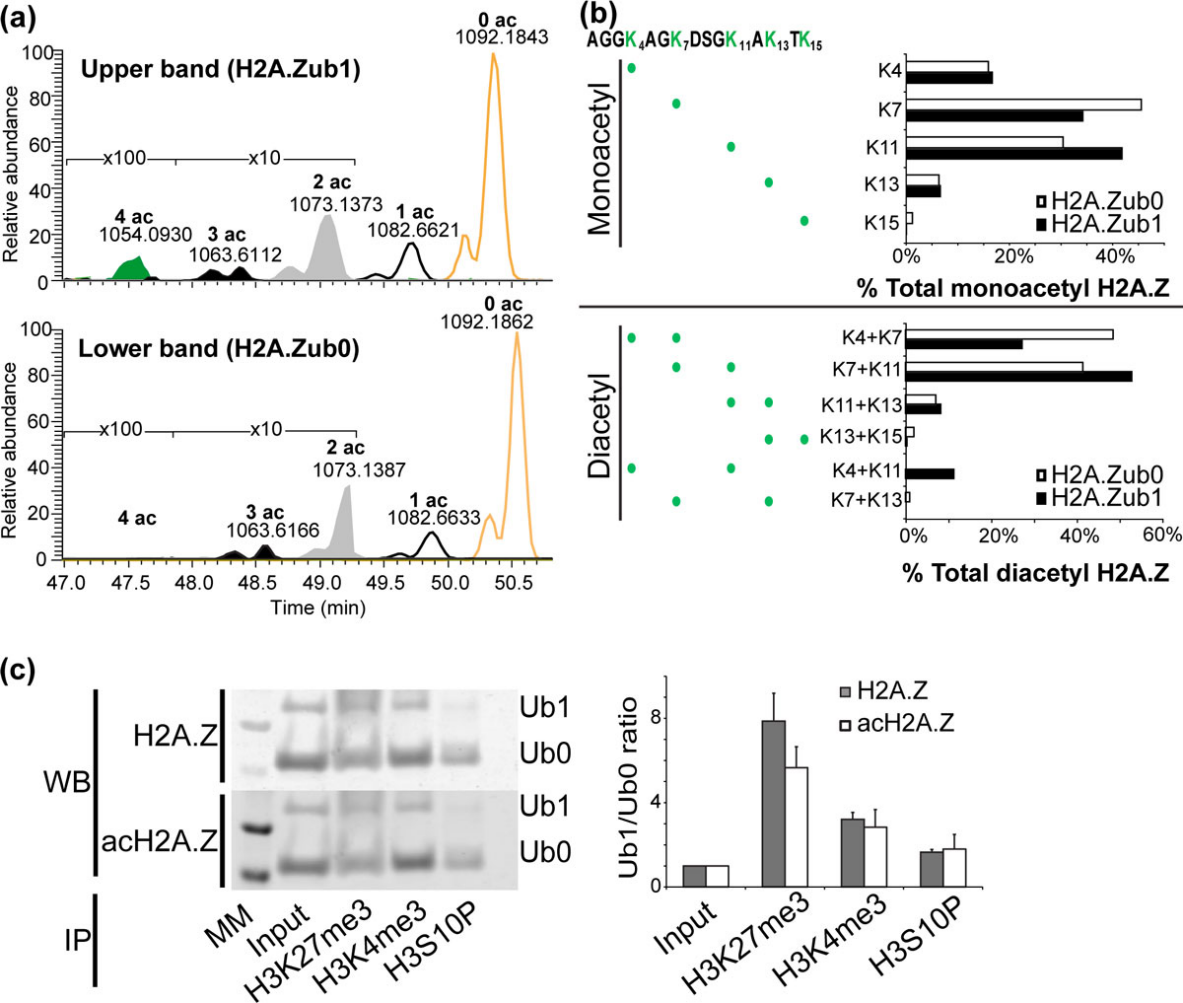
**Dually modified H2A.Z is Ring1B dependent and enriched in bivalent chromatin**. **(a) **Extracted ion chromatograms of upper (H2A.Zub1) and lower (H2A.Zub0) PAGE bands showing presence of various acetyl forms of the N-terminus (1-19) of H2A.Z. **(b) **Site-specific distribution of monoacetyl (top panel) or diacetyl (bottom) moieties on the N-terminal lysines in H2A.Zub0 and H2A.Zub1 fractions. Monoacetylation is preferred on K7 in H2A.Zub0, while K11 is preferred in H2A.Zub1 fractions. Diacetylation often occurs on lysines proximal to each other. Remarkably, diacetylated K4/K11 is observed and specific for H2A.Zub1 fractions. **(c) **Left: western blot image shows the relative levels of ubiquitinated (Ub1) and non-ubiquitinated (Ub0) species in H2A.Z and acH2A.Z in mononucleosomal fractions enriched by immunoprecipitation with antibodies against H3K27me3, H3K4me3 or phosphorylated histone 3 serine 10 residue (H3S10P). Both H2A.Zub1 and acH2A.Zub1 species are more enriched in the H3K27me3 fraction, relative to H2A.Zub0 and acH2A.Zub0 species. The phosphorylation of H3S10 is a hallmark of mitosis, and it anti-correlates with H2Aub1 and H2A.Zub1 levels, thus serving as a negative control [22,52]. Right: Bar plot shows a quantification of the ratio between ubiquitinated and non-ubiquitinated species in H2A.Z (black) and acH2A.Z (white) for each lane. The data are an average of triplicate experiments, normalized by input. Error bars indicate standard deviation. Ac: acetylated; H3S10P: phosphorylated histone 3 serine 10 residue; IP: immunoprecipitation; me3: trimethylation; Ub0: non-ubiquitinated; Ub1: ubiquitinated; WB: western blot.

To decipher further H2A.Z acetylation patterns in relation to ubiquitination status, we used MS to measure site-specific acetylation levels for a population of H2A.Z isolated from mES cells. We characterized the levels and numbers of lysine residues (K4, K7, K11, K13, K15) that are acetylated per molecule (Figure 4a). The most abundant N-terminal H2A.Z acetylation occurs on any one lysine (1ac, 9% in H2A.Zub0 versus 16% in H2A.Zub1), followed by acetylation on any two lysines (2ac, diacetylation, 4% in both H2A.Zub0 and H2A.Zub1). Acetylation on three lysines (3ac, triacetylation) and four lysines (4ac, tetra-acetylation) exists at very low levels (Figure 4a). This analysis also revealed different combinations of acetylation marks occurring at K4, K7, K11, K13 or K15. Interestingly, whereas K14 is the most common acetylation site on yeast H2A.Z, we find that K7 and K11 are the most frequently modified positions for the mammalian variant (Figure 4b). The combinatorial acetylation patterns for H2A.Zub0 and H2A.Zub1 are very similar. For H2A.Zub0 species, the slightly preferred monoacetylated and diacetylated residues are K7, and K4 combined with K7 (K4+K7), respectively. For H2A.Zub1 species, K11 and K7 combined with K11 (K7+K11) appear to be preferred (Figure 4a,b).

### Dually modified H2A.Z enriched within bivalent chromatin in mouse embryonic stem cells

We sought to confirm that the ubiquitinated and dually modified species also localize to bivalent domains. However, we were unable to acquire high-quality ChIP-Seq maps using an antibody against H2A.Zub1. We therefore implemented an alternative approach in which we immunoprecipitated mononucleosomes from mES cells using an antibody against H3K27me3, and then used western blots to evaluate H2A.Z. We found that both H2A.Zub0 and H2A.Zub1 are enriched, and that the level of H2A.Zub1 relative to H2A.Zub0 is significantly higher in the H3K27me3 pull-down relative to control input fractions (Figure 4c). We also performed the same experiment for the ubiquitinated and non-ubiquitinated levels in acetylated H2A.Z (acH2A.Z). We found that the ratio between acH2A.Zub1 and acH2A.Zub0 is significantly higher in H3K27me3-enriched mononucleosomal fractions, normalized by input chromatin. Given that the vast majority of sites with H3K27me3 in mES cells also carry H3K4me3 and are thus bivalent, these results suggest that both ubiquitinated (H2A.Zub1) and dually modified H2A.Z (acH2A.Zub1) are enriched at bivalent chromatin.

### H2A.Z acetylation patterns related to transcriptional status

**Figure 5:**
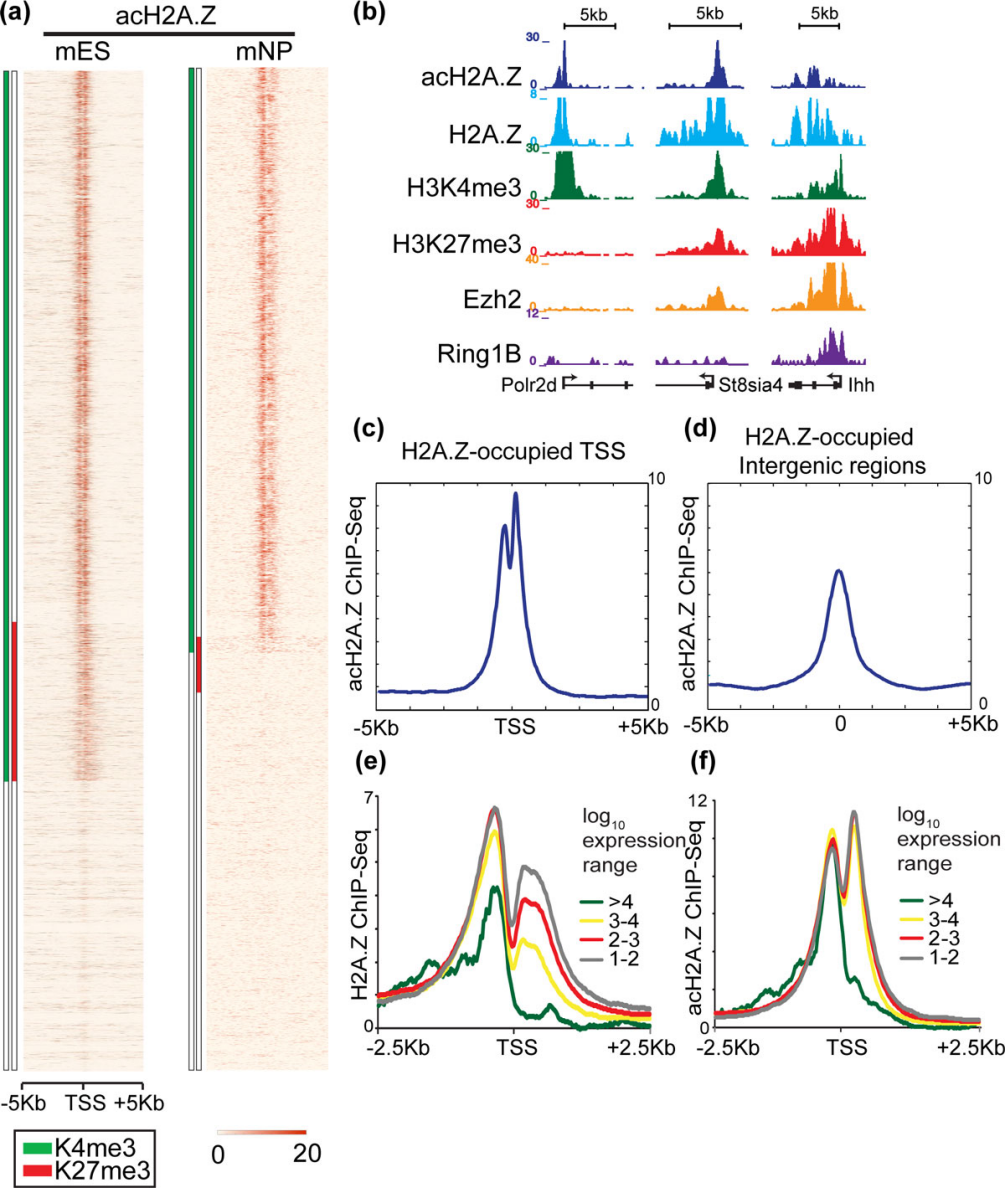
**Acetylated-H2A.Z localizes to bivalent promoters**. **(a) **Heatmaps depict acH2A.Z ChIP-Seq signals at transcription start sites (TSSs) (±5 kb), organized according to their chromatin status: H3K4me3 (green), H3K27me3 (red), bivalent (green and red), and no-mark (white) in mES cells and mNP. acH2A.Z is enriched at promoters in both cell types, but is depleted from monovalent K27me3-only promoters. **(b) **ChIP-Seq tracks show acH2A.Z localization to K4me3-only (Pol2rd) and bivalent promoters (St8sia4 and Ihh) in mES cells. **(c) **Composite plot shows acH2A.Z ChIP-Seq signal is enriched at H2A.Z-enriched TSS (±5 kb). **(d) **Analogous composite plots of acH2A.Z at H2A.Z-enriched intergenic sites (±5 kb). **(e) **Composite plot shows H2A.Z ChIP-Seq signal around TSSs (±2.5 kb), segregated by transcription quartile levels in mES cells. **(f) **Analogous composite plots for acH2A.Z ChIP-Seq signals. ac: acetylated; ChIP-Seq: chromatin immunoprecipitation coupled with high-throughput sequencing; K: lysine; kb: kilobases; me3: trimethylation; mES: mouse embryonic stem; mNP: mouse neural progenitor cell; TSSs: transcription start sites.

Finally, we examined the genomic localization of the acH2A.Z species in mES and mNP cells using ChIP-Seq. We observed clear enrichment for the modified species at bivalent promoters, in addition to actively transcribed TSSs in both mES and mNP cells (Figure 5a). Similar to H2A.Z, acH2A.Z is absent from promoters that only carry H3K27me3 in mNP cells. This suggests that acH2A.Z occupies active and poised promoters, but not stably repressed loci. We also observe strong enrichment of acH2A.Z at both H2A.Z-occupied promoters and intergenic putative enhancers (Figure 5b,c).

Acetylated histones have long been known to be a marker of active transcription and are thought to act in part by neutralizing charge interactions to open chromatin and allow access for transcription machinery [34-37]. To evaluate the correspondence between acH2A.Z levels and transcriptional output, we divided all genes marked by H3K4me3 but without H3K27me3 (H3K4me3-only) into categories according to their expression level. H2A.Z occupancy at the 5' end of transcripts is inversely related to transcriptional activity, similar to published reports (Figure 5d) [13,15]. We specifically found that the most highly active genes in the top expression quartile show lower H2A.Z levels at their 5' ends, possibly due to eviction as a consequence of transcriptional elongation (Figure 5d). Interestingly, acH2A.Z level at the 5' end of the transcript is preserved as transcription level increases (Figure 5e). These data suggest that, as transcription activity escalates, total H2A.Z decreases at the 5' end of the transcript but an increasing proportion of the variant becomes acetylated. The asymmetric distribution of H2A.Z and acH2AZ at active promoters suggests that this histone variant and/or associated chromatin structures may help direct transcription by favoring procession of the transcription machinery towards the 3' direction from TSSs [38].

## Conclusions

Through comprehensive analyses of ChIP-Seq, MS and biochemical data, we document the existence of a novel, dually modified H2A.Z species that preferentially localize to bivalent chromatin domains in ES cells. Our results also clarify that, in both mouse and human ES cells, H2A.Z promiscuously co-localizes to genomic loci enriched for H3K4 methylation, including both bivalent PRC2 targets and as active TSSs as well as at distal enhancer elements. We demonstrate a strong association between H2A.Z acetylation and transcriptional activity. The co-occurrence on the same histone molecule of acetylation, previously linked to gene activity and induction, and ubiquitination events, downstream of Polycomb repressors, echoes the duality of the bivalent H3K4me3 and H3K27me3 combination. We propose that the modified variant contributes to the transcriptional dynamics and epigenomic plasticity of pluripotent ES cells by maintaining dynamic chromatin at key loci poised for alternate developmental fates. This study provides a framework for future studies into the nature of bivalent chromatin functions, and opens up new avenues for decoding the interplay between chromatin-modifying enzymes and modifications of variant histones.

## Materials and methods

### Cell culture

mES cells v6.5 (male, strain *129SVJae *× *C57BL6*) were cultured using standard procedures and reagents [25]. Ring1B KO (*Ring1A^-/-^;Ring1B^fl/fl^; Rosa26::CreERT2*) mES cells have been described previously [39]. mNP cells were *in vitro *differentiated from mES cells as previously described [40]. hES cells (H1) were cultured on Matrigel (BD Biosciences, San Jose, CA, USA) in feeder-free, serum-free modified mTeSR1 media and passaged by dispase digestion (Cellular Dynamics International, Madison, WI, USA) [41].

### Chromatin immunoprecipitation

ChIP experiments for H2A.Z, acH2AZ and other histone modifications were performed in whole cell extract preparation as described previously [25]. The antibody against H2A.Z recognizes both H2A.Z.1 and H2A.Z.2.1. TFIID ChIP was performed by immunoprecipitation of the TFIID subunit TBP (TATA binding protein) in nuclear preparations as detailed previously [25]. Notably, ChIP procedures performed in nuclei preparations were identical to that of whole cell extract, except nuclei were isolated prior to cell lysis and sonication. Crosslinked ES cells were incubated in swelling buffer (0.1 M Tris pH 7.6, 10 mM potassium acetate, 15 mM magnesium acetate, 1% nonyl phenoxypolyethoxylethanol) on ice, and then passed through 16 G needles to disrupt plasma membranes. Nuclei were collected by centrifugation. A summary of ChIP-Seq data sets is listed in Additional file 3 and antibody information is listed in Additional file 4.

### Mass spectrometry

Histones were purified from mES cells as described except that a C5 HPLC column was employed (Phenomenex, Torrance, CA, USA) [42]. Each one-minute fraction collected from the HPLC separation of the histones was subjected to SDS-PAGE. Subsequent LC-MS/MS experiments were performed on an LTQ-Orbitrap mass spectrometer (Thermo Fisher Scientific, Waltham, MA, USA) fed by an Agilent 1100 nano-HPLC system following procedures previously described [43].

Coomassie-stained visible bands from PAGE gels were interrogated by tryptic and chymotryptic digestion. Peptides unique to H2A.Z (H2AFZ or H2A.Z.1, and not derived from other H2A variants) were detected in bands of approximately 14 kDa and approximately 20 kDa that co-purified with the core H4 protein (approximately 12 kDa, see Figure 3c). Next, these bands (from a parallel preparation) were subject to in-gel propionylation using *d_10_*-proprionic anhydride according to [44]. To study the C-terminal ubiquitination of H2A.Z, chymotryptic peptides were analyzed. To study the N-terminal acetylation of H2A.Z, tryptic peptides were analyzed. *M/z *values corresponding to the various acetylated and ubiquitin-residual peptides (recall that the proteases will cleave ubiquitin as well as H2A.Z, leaving a branched peptide residual) were calculated [45]. Separate acquisition methods were designed for the study of acetylation or ubiquitination. Selective-ion monitoring windows were designed around these *m/z*s as appropriate and data-independent MS/MS scans were acquired at these *m/z*s as dictated by each experiment. The sample was introduced to the mass spectrometer via liquid chromatography with conditions identical to those previously described [46].

C-terminal ubiquitinated positional isomers were assigned from collisional MS/MS spectra (Figure 3e). The percentage of each positional isomer was determined using integrated chromatographic peak area of extracted ion chromatograms (Figure 3c, right panel).

N-terminal acetyl positional isomers were also assigned from collisional MS/MS spectra (Figure 3e and data not shown). As positional isomers did not cleanly resolve during chromatographic separation, the percentage of ion current corresponding to *d_5_*-propionylated (non-acetylated) or acetylated b-series fragment ions at each potentially modified residue (K4, K7, K11, K13, K15) in a composite spectrum averaged across the entire elution frame was taken as a proxy for acetylation at that residue. In the case of diacetyl precursors, mixture modeling was conducted to deconvolute percentage occupancy at each individual residue. The total percentage of acetylation was derived from extracted ion chromatograms corresponding to the precursors of the H2A.Z tryptic N-terminus bearing zero to four acetyls (where the balance of primary amines are *d_5_*-propionylated). MS data are available at the Broad Institute Proteomics Platform public data portal [47].

### Western blots

Histones were extracted from mES cells using a standard acid extraction protocol described previously [48]. For the anti-Ring1B western blot, mES cells were incubated with radioimmunoprecipitation assay buffer (BP-115; Boston BioProducts Inc., Ashland MA, USA). Protein concentration was measured by Quant-iT Protein Assay Kit (Q33210; Invitrogen, Carlsbad, CA, USA) to ensure equal loading. Lysate was boiled with NuPage lithium dodecyl sulfate sample buffer (Invitrogen NP0008) and 1% β-mercaptoethanol for 8 minutes before loading on the NuPAGE Novex 4-12% gradient Bis-Tris Gel (Invitrogen NP0322BOX) for electrophoresis. Proteins were transferred using the iBlot system (Invitrogen IB1001) according to manufacturer's instructions. Transferred nitrocellulose membrane was incubated with blocking buffer (Odyssey 927-40000; LI-COR Biosciences, Lincoln, NE, USA), and blotted with respective antibodies overnight. Secondary antibodies conjugated with infrared dye were incubated for 30 minutes in the dark and imaged on the Odyssey^® ^Infrared imaging system (LI-COR Biosciences). For mononucleosome immunoprecipitation, western blot was carried out as described above. Ubiquitinated and non-ubiquitinated bands of H2A.Z and acH2A.Z blots were quantified by using ImageJ software [49].

### Mononucleosome immunoprecipitation

Chromatin preparation and mononucleosome immunoprecipitation of mES cells were performed as described with modifications [22,50]. mES cells were incubated with 0.5% Triton X-100/buffer 1 (10 mM 2-(N-morpholino)ethanesulfonic acid, pH 6.5, 10 mM sodium butyrate, 60 mM potassium chloride (KCl), 15mM sodium chloride (NaCl), 5 mM magnesium chloride (MgCl_2_), 0.25 M sucrose) on ice. Nuclei were then layered onto 2.5× volume of 30% sucrose/buffer 1 and centrifuged at 4,000 rpm. Purified nuclei were resuspended into buffer 2 (20 mM (4-(2-hydroxyethyl)-1-piperazineethanesulfonic acid (HEPES), pH 7.8, 10 mM KCl, 1.5 mM MgCl_2_, 0.34 M sucrose, 10% glycerol, 2 mM calcium chloride, 1 mM dithiothreitol) with 200 U of micrococcal nuclease (10107921001; Roche Diagnostics, Indianapolis, IN, USA) at room temperature until mainly mononucleosomes was achieved, then digestion was stopped by adding a final 1 mM of ethylene glycol tetraacetic acid (EGTA). Digested chromatin was resuspended in buffer 3 (20 mM HEPES, pH 7.8, 1.5 mM MgCl_2_, 0.42 M NaCl and 0.2 mM EGTA), incubated on ice for 1 hour and centrifuged at 1000 g. Finally, 2.8× volume of buffer 4 (20 mM HEPES, pH 7.8, 1.5 mM MgCl_2_, 25% glycerol, 0.2 mM EGTA) was added drop-wise to the supernatant while vortexing.

The supernatant was used for subsequent immunoprecipitation by adding antibodies and incubated at 4°C. Protein-A or -G Sepharose beads (Sigma-Aldrich, St. Louis, MO, USA) were added and incubated for 2 hours then subjected to eight washes of buffer 5 (20 mM HEPES, pH 7.8, 1.5 mM MgCl_2_, 10% glycerol, 0.2% Triton X-100, 150 mM NaCl, 0.2 mM EGTA). Buffers 1 to 5 were supplemented by phenylmethylsulfonyl fluoride and benzamidine (0.1 mM each). Histones were eluted by boiling the washed beads in lithium dodecyl sulfate sample buffer, and 1% β-mercaptoethanol.

### Illumina sequencing

Illumina sequencing libraries were prepared from ChIP-enriched DNA as described previously [7,25], and sequenced on the Illumina Genome Analyzer IIx and HiSeq2000 (Illumina, San Diego, CA) according to the manufacturer's specifications. ChIP-Seq data was compiled, processed and aligned as published [25]. All ChIPs performed in mouse cells were aligned to mm8, and ChIPs in human cells were aligned to hg18 reference genomes.

### Computational analysis

ChIP-Seq data was processed and aligned to the reference genomes (mm8 for mES, mNP and hg18 for hES) as described in a previous study [7]. Promoter classification and ChIP-Seq enriched intervals were carried out as described [7,25]. Promoters were defined as 0.5 kb upstream and 2 kb downstream of all annotated TSSs, generating 17,760 mouse and 18,522 human promoters respectively. Genomewide-enriched windows were calculated in sliding one-kilobase windows and are merged if distance between the two is less than 2 kb. ChIP-Seq enriched intergenic regions are defined as enriched windows that are least ±4 kb from known gene promoters and gene bodies to prevent contamination of proximal or alternate promoters. Heatmaps were generated by measuring ChIP-Seq signals in 200 bp sliding windows spanning ±5 kb of the TSS. Composite plots were generated by averaging values in each of the 200 bp windows. Statistical significance of enrichment (*P *< 10^-4^) was determined based on background distribution of randomized reads specific for each independent genomewide ChIP analysis. MTLs were defined previously as described [28]. Chromosome positions for MTLs were extended ±2 kb. MTLs that are located in ±4 kb of TSSs and transcription end sites, or gene bodies were removed to yield intergenic MTLs, and query the overlap with H2A.Z defined genomewide-enriched windows (mentioned above). mES cell mRNA enrichment analysis was generated using published RNA-Seq data [30], and a heatmap was generated as described above. ChIP-seq data are available at the Broad Institute Epigenomics Public Data Portal [51].

### Accession number

The data sets are available in the Gene Expression Omnibus (GEO) database under the accession number GSE:[39237].

## Abbreviations

acH2A.Z: acetylated H2A.Z; acH2A.Zub0: acetylated and non-ubiquitinated H2A.Z; acH2A.Zub1: acetylated and monoubiquitinated H2A.Z; bp: base pair; ChIP-chip: chromatin immunoprecipitation coupled with microarray; ChIP-Seq: chromatin immunoprecipitation coupled with high-throughput sequencing; EGTA: ethylene glycol tetraacetic acid; ES: embryonic stem; H2A.Zub0: non-ubiquitinated H2A.Z; H2A.Zub1: monoubiquitinated H2A.Z; H3: histone H3; H3K4me3: histone 3 lysine 4 trimethylation; H3K27me3: histone 3 lysine 27 trimethylation; HEPES: 4-(2-hydroxyethyl)-1-piperazineethanesulfonic acid; hES: human embryonic stem; HPLC: high performance liquid chromatography; K: lysine; kb: kilobases; KCl: potassium chloride; LC: liquid chromatography; me1: monomethylation; me2: dimethylation; me3: trimethylation; mES: mouse embryonic stem; MgCl_2_: magnesium chloride; mNPs: mouse neural progenitors; MS: mass spectrometry; MTLs: multiple transcription factor binding loci; NaCl: sodium chloride; PRC: Polycomb repressive complexes; RNAPII: RNA polymerase II; TF: transcription factor; TFIID: transcription Factor II D; TSSs: transcription start sites.

## Competing interests

The authors declare that they have no competing interests.

## Authors' contributions

MK conceived the study, performed the experiments, analyzed the data and wrote the manuscript. JJ performed and analyzed the mass spectrometry experiments. RK and ER analyzed data. ME and HK generated the Ring1B knockout ES cells. SC coordinated mass spectrometry experiments. BB conceived the study, participated in its design and helped to draft the manuscript. All authors read and approved the final manuscript.

## Supplementary Material

Additional file 1 **Composite plots show H3.3 ChIP-Seq signal enriches at K4me3-only and bivalent promoters (PRC1-positive (+) and PRC1-negative (-)), but is depleted in no-mark promoters in mES cells**. Click here for file

Additional file 2 **Composite plots show H3 distribution across K4me3-only, bivalent (PRC1-positive and PRC1-negative) or no-mark TSSs (±2.5 kb)**. Nucleosome-deficient regions are observed at K4me3-only and bivalent promoters, but not at no-mark promoters. Click here for file

Additional file 3 **Summary of ChIP-Seq data sets**. Click here for file

Additional file 4 **Summary of antibody information**. Click here for file
