# Supplementary figures and images for: H2A.Z landscapes and dual modifications in pluripotent and multipotent stem cells underlie complex genome regulatory functions
Source: Genome Biol. 2012 Oct 3;13(10):R85. doi: 10.1186/gb-2012-13-10-r85 (PMC3491413; doi:10.1186/gb-2012-13-10-r85)

# mES cells

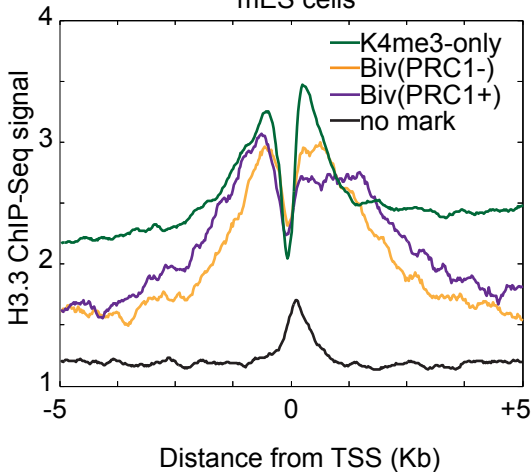

Supplement: Additional file 1 — Composite plots show H3.3 ChIP-Seq signal enriches at K4me3-only and bivalent promoters (PRC1-positive (+) and PRC1-negative (-)), but is depleted in no-mark promoters in mES cells. [file gb-2012-13-10-r85-S1.pdf]

## mES cells

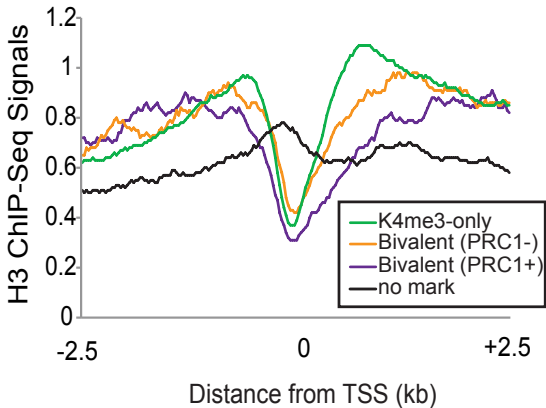

Supplement: Additional file 2 — Composite plots show H3 distribution across K4me3-only, bivalent (PRC1-positive and PRC1-negative) or no-mark TSSs (±2.5 kb). Nucleosome-deficient regions are observed at K4me3-only and bivalent promoters, but not at no-mark promoters. [file gb-2012-13-10-r85-S2.pdf]
