# Supplementary material for: H2A.Z landscapes and dual modifications in pluripotent and multipotent stem cells underlie complex genome regulatory functions
Source: Genome Biol. 2012 Oct 3;13(10):R85. doi: 10.1186/gb-2012-13-10-r85 (PMC3491413; doi:10.1186/gb-2012-13-10-r85)
Supplement: Additional file 3 — Summary of ChIP-Seq data sets. [file gb-2012-13-10-r85-S3.pdf]

Cell Type	Antibody	Uniquely aligned Reads
mES	H2A.Z	11954153
	AcH2A.Z	6566755
	TFIID (TBP)	5938208
	RNAPII (CTD4H8)	3276242
mNP	H2A.Z	13629978
	acH2A.Z	974569
hES	H2A.Z	22469450
