# Supplementary material for: H2A.Z landscapes and dual modifications in pluripotent and multipotent stem cells underlie complex genome regulatory functions
Source: Genome Biol. 2012 Oct 3;13(10):R85. doi: 10.1186/gb-2012-13-10-r85 (PMC3491413; doi:10.1186/gb-2012-13-10-r85)
Supplement: Additional file 4 — Summary of antibody information. [file gb-2012-13-10-r85-S4.pdf]

Antibody	Source	Application
H2A.Z	Millipore 07-594	ChIP, WB
AcH2A.Z	Abcam ab18262	ChIP, WB
TFIID (TBP)	Santa Cruz sc-273X	ChIP
RNAPII (CTD4H8)	Covance MMS-128P	ChIP
H3K4me3	Abcam ab8580	ChIP, MN-IP
H3K4me2	Abcam ab7766	ChIP
H3K4me1	Abcam ab8895	ChIP
H3K27me3	Millipore 07-449	ChIP, MN-IP
Ring1B	MBL D139-3	WB
H3S10P	Cell Signaling 9706S	MN-IP
IRDye® 800CW Goat Anti-mouse IgG	Odyssey® 926-32212	WB
IRDye® 680CW Goat Anti-rabbit IgG	Odyssey® 926-32223	WB
